# Hepatocellular carcinoma cell-derived small extracellular vesicle-associated CD147 serves as a diagnostic marker and promotes endothelial cell angiogenesis via the PI3K/Akt pathway

**DOI:** 10.20517/evcna.2023.30

**Published:** 2023-10-12

**Authors:** De-Fa Huang, Wen-Juan Zhang, Jie Chen, Zhi-Gang Jiao, Xiao-Ling Wang, Ding-Yu Rao, Wei-Song Li, Die Hu, Fang-Fang Xie, Xiao-Xing Wang, Zheng-Zhe Li, Xiao-Mei Yi, Ji-Yang Wu, Yu Jiang, Qi Wang, Tian-Yu Zhong

**Affiliations:** ^1^Laboratory Medicine, First Affiliated Hospital of Gannan Medical University, Ganzhou 341000, Jiangxi, China.; ^2^Precision Medicine Center, First Affiliated Hospital of Gannan Medical University, Ganzhou 341000, Jiangxi, China.; ^3^Department of Clinical Laboratory, Yongchuan Hospital of Chongqing Medical University, Chongqing 402160, Sichuan, China.; ^4^Department of Cardiothoracic Surgery, First Affiliated Hospital of Gannan Medical University, Ganzhou 341000, Jiangxi, China.; ^5^Department of pathology, First Affiliated Hospital of Gannan Medical University, Ganzhou 341000, Jiangxi, China.; ^6^The First School of Clinical Medicine, Gannan Medical University, Ganzhou 341000, Jiangxi, China.; ^7^Department of Pharmacology and Chemical Biology, University of Pittsburgh School of Medicine, Pittsburgh, PA 15260, USA.; ^#^These authors contributed equally and considered joint first authors.

**Keywords:** Hepatocellular carcinoma, small extracellular vesicles, CD147, diagnosis, angiogenesis

## Abstract

**Aim:**

Hepatocellular carcinoma (HCC) is one of the most common malignant tumors. The process of HCC development is closely related to angiogenesis. Plasma exosomes have diagnostic value in many diseases and have become a current research hotspot. We aimed to identify a key molecule in small extracellular vesicles (sEVs) involved in angiogenesis as a diagnostic marker for HCC and uncover the mechanism underlying its regulation in the angiogenesis process.

**Methods:**

Nano‐flow cytometer (nFCM) was used to detect CD147 expression in plasma-derived sEVs in 155 HCC patients, 59 liver cirrhosis (LC), and 82 healthy donors (HD). The mechanism of hepatocellular carcinoma cell-derived sEVs CD147 promoting angiogenesis was elucidated by cell proliferation assay, scratch wound healing assay, transwell assay, tube formation assay, and *in vivo* Matrigel plug angiogenesis assay.

**Results:**

We found that CD147 expression was significantly higher in HCC tissue samples than in normal tissues. We also found a significantly larger number of CD147-positive small extracellular vesicles (CD147^+^ sEVs) in the plasma of HCC patients than LC patients and HD. Furthermore, we showed that hepatocellular carcinoma cell (HepG2)-derived CD147^+^ sEVs promoted cell proliferation, migration, invasion, and angiogenesis in human umbilical vein endothelial cells. The CD147^+^ sEVs upregulated vascular endothelial growth factor A (VEGFA) by activating the PI3K/Akt pathway, thereby promoting angiogenesis.

**Conclusion:**

HCC-derived sEVs-associated CD147 serves as a diagnostic marker and promotes endothelial cell angiogenesis via the PI3K/Akt pathway.

## INTRODUCTION

HCC is the most common primary liver cancer, accounting for approximately 90% of all liver cancer cases^[1]^. According to the World Health Organization, HCC is currently the fifth most common cancer worldwide and the third leading cause of cancer-related mortality^[2]^. HCC is a common malignant tumor with a rich blood supply, and its development process is closely linked to angiogenesis. Angiogenesis not only promotes tumor growth but also facilitates the shedding of tumor cells. These enter the blood vessels or spread to the adjacent stroma, creating conditions for the invasion and metastasis of HCC^[3]^. Currently, screening for HCC is based on the measurement of serum alpha-fetoprotein (AFP), imaging techniques, and histology^[4,5]^. However, in patients with atypical AFP levels, the AFP value lacks sufficient sensitivity and specificity for predicting HCC. In addition, imaging quality is inadequate for the diagnosis of tumors smaller than 1 cm in diameter. Although tissue biopsy can be accurate, it is an invasive procedure that may increase the incidence of needle metastasis^[6]^. Therefore, uncovering the key regulatory factors of HCC angiogenesis would provide novel diagnostic and therapeutic targets and aid in the development of new HCC drugs.

The different cell types in the tumor microenvironment not only function through direct contact and production of soluble factors but also release extracellular vesicles (EVs) to regulate angiogenesis^[7]^. The term EVs collectively refers to nanoscale vesicles with membrane structures that are actively secreted by cells^[8]^. In the past, EVs were classified into exosomes, microvesicles, and apoptotic vesicles, depending on their formation mechanism. However, as there is no specific marker molecule to distinguish between different types of EVs, the International Society for Extracellular Vesicles (ISEV) suggested the division of EVs into small EVs (sEVs) (diameter less than 200 nm) and large EVs (lEVs) (diameter greater than 200 nm)^[9]^.

Non-coding RNAs, such as miRNA, lncRNA, and circRNA, are cargo molecules within hepatocellular carcinoma cell-derived sEVs that have been identified in previous studies. Recently, it was shown that surface membrane structures encasing EVs contain a large cohort of active pro-angiogenic proteins^[10]^. Membrane proteins on the surface of sEVs can bind to target cell receptors as ligands or can be endocytosed by target cells to stimulate the vascular neointimal cascade response^[11]^.

Cluster of differentiation 147 (CD147), also known as extracellular matrix metalloproteinase inducer (EMMPRIN) or basigin (BSG), is a type I transmembrane glycoprotein and a member of the immunoglobulin superfamily^[12]^. CD147 is expressed at a high level on the surface of many types of tumor cells, including hepatocellular carcinoma and breast cancer. This protein regulates tumor cell proliferation, invasion, metastasis, angiogenesis, chemotherapy resistance, and energy metabolism^[13-15]^. It was shown that ovarian cancer cell-derived lEVs stimulated human umbilical vein endothelial cells (HUVECs) to produce matrix metallopeptidases (MMPs) through CD147 and induced angiogenesis^[16]^. In addition, recombinant soluble CD147 extracellular segment has been shown to bind to the CD147 receptor on the surface of fibroblasts, liver cancer cells, and breast cancer cells. This led to the activation of PI3K/Akt, ERK, FAK, and Wnt/β-catenin to induce the production of MMPs, CD147, and VEGF^[17-19]^. In the current study, we found that the secretion of CD147-positive sEVs was significantly increased in hepatocellular carcinoma. We also demonstrated that hepatocellular carcinoma-derived sEVs promoted angiogenesis through CD147.

## MATERIALS AND METHODS

### Mining of the cancer genome atlas database

CD147 expression data (fragments per kilobase per million mapped reads, FPKM) from 50 pairs of mining of the cancer genome atlas (TCGA) clinical breast cancer and normal breast tissues were downloaded from the Genomic Data Commons (GDC) Data Portal. The heatmap was visualized using Java TreeView. CD147 expression data (transcripts per kilobase per million mapped reads, TPM) in a cohort of TCGA clinical breast samples (tumor: 369; normal: 50) were downloaded from the GDC Data Portal. Box plots and violin plots with significance were generated using TCGA samples as input using Gene Expression Profiling Interactive Analysis (GEPIA, http://gepia.cancer-pku.cn/).

### Clinical samples and plasma preparation

The blood samples were obtained from the First Affiliated Hospital of Gannan Medical University. 59 pathologically confirmed LC patients, 155 HCC patients, and 82 HD were enrolled between January 2021 and July 2021. All patients underwent magnetic resonance imaging, abdominal ultrasound, and CT examinations to identify clinical symptoms and signs. Serum AFP levels were also measured. We used 10 ng/mL as the cutoff value for AFP. Clinical characteristics were obtained from the electronic medical record. Tumor staging was performed according to the Barcelona Clinic Liver Cancer(BCLC) staging system. Written informed consent was obtained from all donors. This study was approved by the Ethics Committee of the First Affiliated Hospital of Gannan Medical University (No.2022185). The blood samples were collected from donors using EDTA anticoagulation tubes and centrifuged at 1,500 *g* for 20 min to remove the cells from the blood. The supernatant was centrifugated at 3,000 *g* for 15 min to collect the plasma, which was stored at -80 °C.

### Cell culture and lentiviral infection

HepG2 cell line and the human umbilical vein endothelial cell line (HUVEC) were obtained from the Cell Bank of Chinese Academy of Sciences (Shanghai, China). HepG2 and HUVECs were cultured in DMEM medium (Gibco, USA) containing 10% fetal bovine serum (FBS, Excell Bio, Uruguay) and 1% penicillin/streptomycin (Solarbio, China). The cells were maintained in cell culture dishes in a humidified chamber at 37 °C with 5% CO_2_. HUVECs were treated with MK-2206 (Akt inhibitor, Apexbio, USA) at a concentration of 1 μM for 24 h. Full-length human CD147 (NM_001728) cDNA was cloned into the pCDH-CMV-MCS-EF1-Puro lentiviral vector (Shanghai Genechem Co., Ltd.). Short hairpin RNA (shRNA) targeting CD147 (shCD147, CCGGTCAGAGCTACACATTGA) was cloned into the pLKO.1 lentiviral vector. The CD147 knockdown cell line was established by lentiviral infection. HepG2 cells (5 × 10^4^) were inoculated in 6-well plates and the lentivirus added for 16 h after the cells adhered to the wall. After 72 h, puromycin (2 μg/mL) screening was applied to obtain the CD147 stable knockdown cell line HepG2.

### Isolation of sEVs

To isolate sEVs, plasma was centrifuged at 10,000 *g* for 30 min to remove cell debris, followed by ultracentrifugation at 110,000 *g* for 64 min to pellet the sEVs (Beckman, USA). Finally, the sEVs were washed with phosphate‐buffered saline (PBS) and pelleted again by ultracentrifugation at 110,000 *g* for 64 min. The sEVs pellet was resuspended in 100 µL of PBS for subsequent use. All centrifugations were performed at 4 °C.

FBS was centrifuged at 100,000 *g* for 16 h at 4 °C followed by filtering with a 0.22 μm pore filter (Millipore, Germany) to prepare ‘EV-depleted FBS’^[20]^; the cells were cultured in DMEM with 10% ‘EV-depleted FBS’ for 2 days until 80%-90% confluence. 50 mL of cell culture medium was collected and centrifuged at 1,000 *g* for 10 min to remove cell debris, followed by centrifugation at 10,000 *g* for 20 min to remove large vesicles. The supernatant was further centrifuged at 110,000 *g* for 50 min to pellet the sEVs (Beckman, USA). Finally, the sEVs were washed with PBS and pelleted again by ultracentrifugation at 110,000 *g* for 50 min. The sEVs pellet was resuspended in 100 µL of PBS for subsequent analyses. All centrifugations were performed at 4 °C.

### Transmission electron microscopy imaging

A volume of twenty microliters from the sEVs sample was placed on a copper grid for 1 min. The sEVs-coated copper grid was stained with 20 μL of 2% uranyl acetate staining solution for 1 min. The stained copper grid was baked for 3 min under a 60 W incandescent lamp and examined by transmission electron microscope (TEM) (JEOL, Japan).

### Western blotting

Proteins were separated by 12% SDS-containing polyacrylamide gel electrophoresis and transferred to a polyvinylidene fluoride microporous membrane. The membrane was blocked with 5% skim milk powder for 1 h, followed by overnight incubation at 4 °C with antibodies to CD9 (Abcam, USA, ab263019), CD63 (Abcam, USA, ab134045), TSG101 (Abcam, USA, ab125011), GM130 (Abcam, USA, ab52649), CD147 (Abcam, USA, ab108308). AKT1 (Abcam, USA, ab81283), phosphorylation-AKT1 (ABS, USA, S473), VEGFA (Abcam, USA, ab214424), and GAPDH (Proteintech, China, 60004-1-Ig). Horseradish peroxidase-labeled goat anti-rabbit/mouse IgG antibody (Proteintech, China, A00001-2 /SA00001-1) was used as the secondary antibody.

### Nanoparticle flow cytometry analysis

Particle concentration, particle size distribution, and surface proteins of the sEVs samples were analyzed by nano‐flow cytometer (nFCM). For particle size, a particle size standard curve was obtained using silica nanoparticle size standards on an nFCM detector. The particle size distribution was obtained using 20 μL of sEVs sample. For surface protein detection, FITC-coupled mouse anti-human CD147 antibody (4A Biotech, China, FHF147-025) was used. The sEVs (50 μL) were mixed with 5 μL of anti-CD147 antibody and incubated for 30 min at 37 °C. After incubation, sEVs were washed twice by centrifugation at 4 °C for 30 min at 110,000 *g*. After the final wash, the sEVs pellet was resuspended in 50 μL of PBS, and the proportion of CD147 positive sEVs was obtained using the nFCM system. We identified the cutoff value of CD147^+^ sEVs (%) as 12.4 based on the sum of sensitivity and 1- specificity in AFP-negative patients.

### PKH67-labeled sEVs

The isolated sEVs were labeled with a PKH67 green fluorescent labeling kit (Sigma, USA) following the manufacturer’s instructions. HUVECs were seeded in 6-well plates. After the cells were completely adherent, membrane dye (prepared with the medium at a ratio of 1:1000) was added for staining at 37 °C for 15 min. Then, 100 μL of sEVs and 4 μL of PKH67 were diluted in 1 mL of Dilution C, and then mixed evenly. The mixture was incubated for 4 min at room temperature. Then, 2 mL of 1% bovine serum albumin (BSA) was added to bind PKH67. The sample was centrifuged twice at 110,000 *g* for 70 min to remove the excess PKH67. PKH67-labeled sEVs were resuspended in DMEM medium and added to the HUVECs. After co-incubation for 12 h, the cells were fixed with 2% paraformaldehyde for 10 min and then blocked with mounting medium (with DAPI). Images were taken with a confocal fluorescence microscope (ZEISS, Germany).

### Immunohistochemistry

Formaldehyde-fixed and paraffin-embedded sections of tissue were subjected to immunohistochemistry and stained with monoclonal antibodies against CD147 (Abcam, USA, ab194401) or CD31 (Abcam, USA, ab182981) using a streptavidin-peroxidase staining kit (Proteintech, China). The percentage of positive cells was graded on a scale of 0 to 4 (0: negative, 1: 0%-25%, 2: 26%-50%, 3: 51%-75%, 4: 76%-100%). Positive areas were further quantified from 0 to 3 using the relative staining intensity (1: weak, 2: moderate, 3: intensive). The final score was obtained by multiplying the intensity by the quantification measurements. Images were obtained using a fully automated digital pathology slide scanner. (KFBIO, China).

### Cell proliferation assay

HUVECs (5 × 10^3^ cells per well; five replicates per group) were seeded into 96-well culture plates and treated with sEVs (100 μg/mL) from different groups or PBS. A group without cells served as the blank control. After 24 h, cell counting kit-8 (CCK-8) reagent (10 μL per well; Biosharp, China) was added to the culture medium (100 μL per well). After incubation at 37 °C for 1.5 h, the absorbance of each well was measured at 450 nm using a microplate reader.

### Scratch wound healing assay

HUVECs (5 × 10^5^ cells per well; three replicates per group) were seeded into 6-well plates and incubated at 37 °C until the cells had attached. Linear scratch wounds in the cell monolayer were created with a sterile 200 μL pipet tip. The wells were washed with PBS to remove detached cells and then exposed to sEVs (100 μg/mL) from different groups or an equal volume of PBS. HUVECs were photographed at 0 h and 48 h after wounding. Migration rate (%) = (wound area after 0 h-wound area after 48 h)/wound area after 0 h × 100%.

### Transwell assay

For the transwell assay, 5 × 10^4^ cells/well (three replicates per group) were suspended in serum-free medium and seeded into the upper chamber of 24-well transwells (Corning, USA) containing 8 μm pore filters. Then, cells were treated with sEVs (100 μg/mL) from different groups or PBS. Complete medium (650 μL) containing 15% FBS was added to the lower chamber. After 24 h, the cells that had attached to the upper surface of the filter membranes were removed, and the migrated cells on the lower surface were stained with 0.1% crystal violet for several minutes. The cells were photographed under an optical microscope.

### Tube formation assay

Cold Matrigel (BD, USA) (50 ml/per well) was transferred into each well of a 96-well plate and incubated at 37 °C for 30 min. Then, HUVECs (1 × 10^4^ cells per well; three replicates per group) were plated into the Matrigel-coated 96-well plates and treated with sEVs (100 μg/mL) from different groups or PBS. Six hours after seeding, images of tube formation were captured under an inverted microscope.

### *in vivo* matrigel plug angiogenesis assay

Female athymic‐nude (nu/nu) mice (7-8 weeks old) were purchased from Slac Laboratory Animal Co., Ltd. (Hunan, China). Four groups (5 mice per group) were randomly allocated into groups for the *in vivo* matrigel plug assay: Matrigel+HUVECs+PBS, Matrigel+HUVECs+HepG2-WT-sEVs, Matrigel+HUVECs+HepG2-CD147(KD)-sEVs, Matrigel+MK-2206 dihydrochloride-treated HUVECs+HepG2-WT-sEVs. To test whether different groups of sEVs affected HUVECs angiogenesis, 5 × 10^6^ HUVECs were mixed with 500 μL of Matrigel (BD, USA) at a ratio of 1:1, and sEVs (100 μg/mL) from different groups or PBS were resuspended in 500 μL of ice-cold Matrigel. The above groups were implanted subcutaneously on the backs of nude mice. Matrigel plugs were removed for analysis 14 days later. Plug volume= (width^2^ × length)/2.

### Statistical analysis

All quantitative data are presented as means ± SE. Comparison between the two groups was performed using Student’s *t* test. Statistical analyses of data between multiple groups were performed using one-way analysis of variance (ANOVA). The scatter plots were generated using GraphPad Prism 8.0. Flow plots were generated using Flowjo 10.6. The paired comparison of receiver operating characteristic (ROC) curves was conducted using MedCalc version 18.0. Statistical significance was set at *P* < 0.05.

## RESULTS

### High-level expression of CD147 is associated with HCC

**Figure 1:**
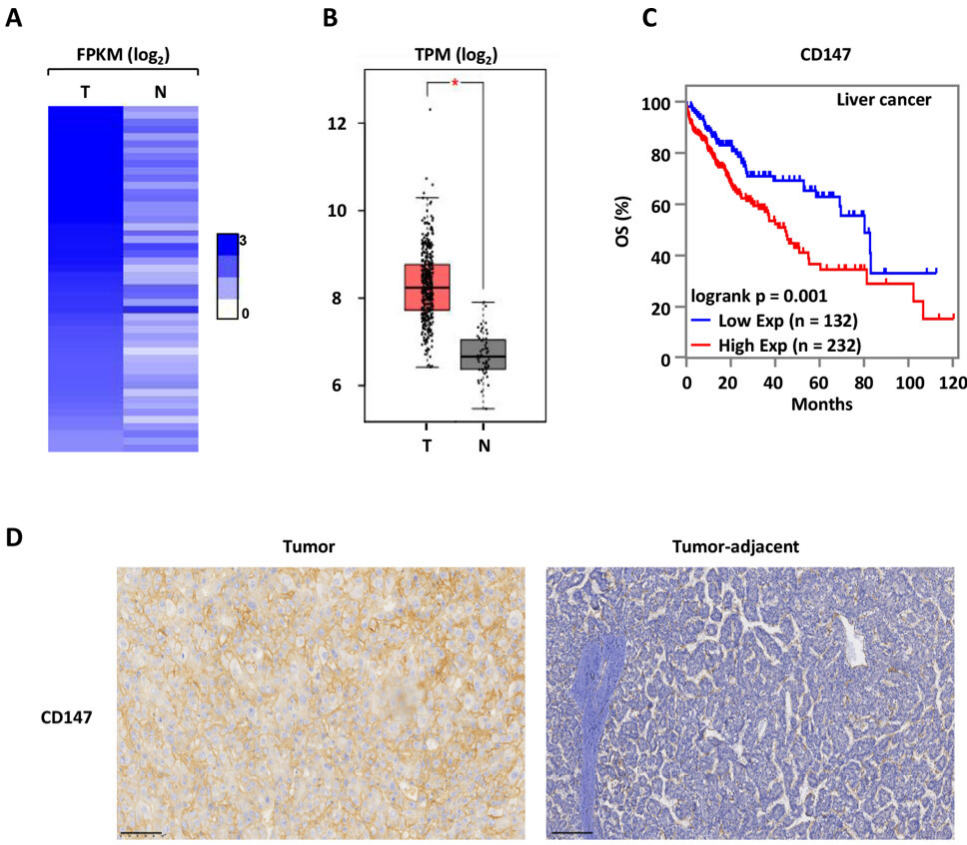
High-level expression of CD147 is associated with liver tumorigenesis. (A) The expression levels (FPKM, log_2_) of CD147 in tumor tissues (*n* = 50) of clinical liver cancer and normal tissue samples from TCGA; (B) The expression (TPM, log_2_) of CD147 in a cohort of clinical liver cancer (*n* = 369) and normal (*n* = 50) samples from TCGA; (C) Kaplan Meier survival analyses for OS; (D) The expression of CD147 in HCC tumor and tumor-adjacent samples (*n* = 22) was analyzed by immunohistochemistry. Representative images are shown. Scale bar: 100 μm. TCGA: the cancer genome atlas; OS: overall survival; HCC: hepatocellular carcinoma.

High-level expression of CD147 was found in liver samples of HCC patients in comparison to normal liver tissues [Figure 1A and B] and correlated positively with the cancer progression stage [Supplementary Figure 1A]. Furthermore, high expression of CD147 was correlated with poor prognosis in liver cancer patients [Figure 1C and Supplementary Figure 1B-D]. Immunohistochemical analyses also showed that the expression of CD147 in tumor tissues was higher than in tumor-adjacent tissues [Figure 1D].

### Plasma CD147^+^ sEVs are clinically relevant

**Figure 2:**
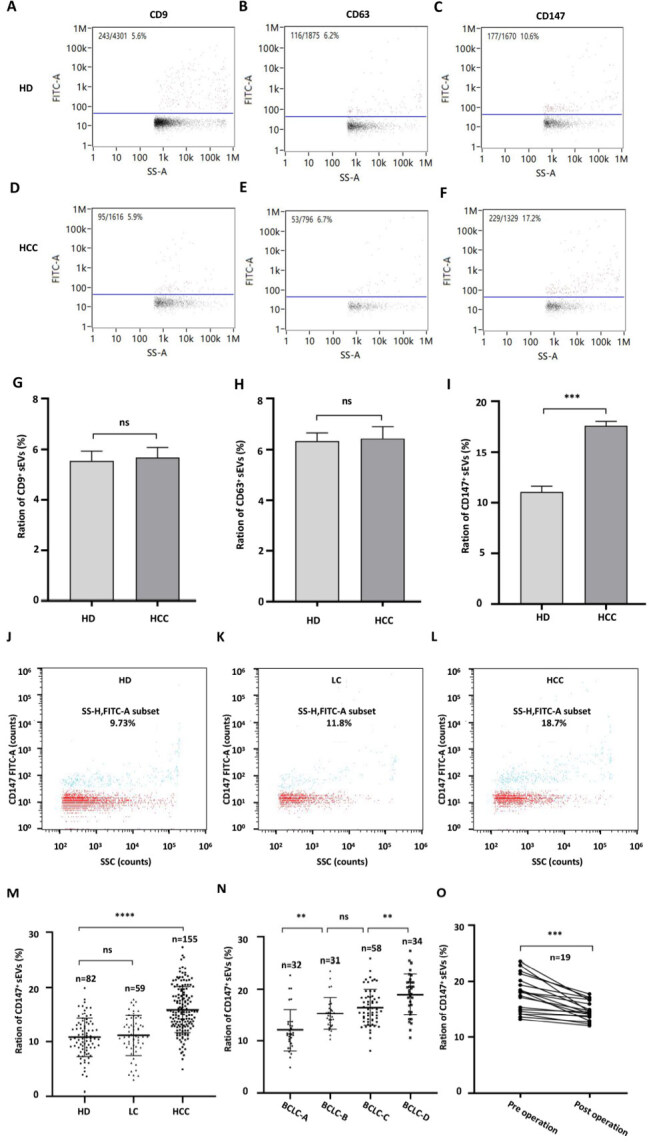
Plasma levels of CD147^+^ sEVs are clinically relevant. (A-F) The bivariate dot plots of CD9(A, D), CD63(B, E) and CD147(C, F) FITC fluorescence (y-axis) *vs*. SS-A (x-axis) for sEVs isolated from plasmas of in HD (A-C) and HCC patients (D-F); (G) The ratios of CD9^+^ sEVs *vs*. total sEVs (CD9^+^/total) were not significantly different between HD and HCC groups; (H) The ratios of CD63^+^ sEVs *vs.* total sEVs (CD63^+^/total) were not significantly different between HD and HCC groups; (I) The ratios of CD147^+^ sEVs *vs*. total sEVs (CD147^+^/total) were significantly different between HD and HCC groups; (J-L): (J) The bivariate dot plots of CD147 FITC fluorescence (y-axis) *vs*. SSC (x-axis) for sEVs isolated from plasmas of HD; (K) LC patients; (L) HCC patients; (M) The ratios of CD147^+^ sEVs *vs*. total sEVs (CD147^+^/total) were significantly different between HD and LC/HCC groups; (N) The changes in the ratio of CD147^+^/total sEVs were significant between BCLC stages; (O) The ratio of CD147^+^/total sEVs in the plasma of HCC patients (*n* = 19) before (pre-operation) and 7-10 days after (post-operation) surgical removal of the tumors. ns: not significant; **: *P* < 0.01; ***: *P* < 0.001; ****: *P* < 0.0001; HCC: hepatocellular carcinoma; CD147^+^ sEVs: CD147-positive small extracellular vesicles; HD: healthy donors; LC: Liver cirrhosis; BCLC: Barcelona Clinic Liver Cancer.

nFCM analysis of sEVs isolated from plasma showed that sEVs size (Plasma-sEVs) ranged from 50 to 180 nm [Supplementary Figure 2A]. In addition, the morphology exhibited elliptical membranous vesicles, as observed by TEM [Supplementary Figure 2B]. As expected, protein markers commonly associated with sEVs, such as CD9, CD63, and TSG101, were present in the sEVs, and the negative EVs marker GM130 (the Golgi protein) was not detected [Supplementary Figure 2C]. To demonstrate that particles detected by nFCM assays are actual sEVs, we simultaneously tested the positive rates of CD9, CD63, and CD147. We found no difference in the rate of CD9 or CD63 between HCC and HD, while there was a significant difference in CD147 [Figure 2A-I]. This confirms that our detection of CD147 in sEVs is credible and effective. Analysis of plasma sEVs showed that CD147^+^ sEVs levels were significantly higher in the HCC group compared with the HD and LC groups [Figure 2J-M]. The levels of CD147^+^ sEVs also increased with increasing BCLC stage [Figure 2N]. Most importantly, blood samples analyzed before and after surgery revealed that 19 HCC patients exhibited a significant decrease in CD147^+^ sEVs levels after surgical resection [Figure 2O].

The relationship between plasma CD147^+^ sEVs levels and clinical characteristics in HCC patients is shown in Table 1. The original information on HD, LC patients and HCC patients is shown in Supplementary Tables 1-3. Plasma CD147^+^ sEVs levels were not statistically different with age, gender, HBV infection, liver cirrhosis, and number of tumors. However, the levels correlated strongly with cancer stage, and statistically significant differences in relation to tumor size, portal vein tumor thrombus (PVTT), extrahepatic metastasis (EHM), AFP, and BCLC staging were observed. A significant increasing trend was observed in the level of CD147^+^ sEVs and cancer progression.

**Table 1:** Associations between plasma sEVs-CD147 level and clinical characteristics of HCC patients

**Clinico-pathological characteristics**		**N (155)**	**CD147^+^ sEVs (%)**	***P***
Age (years)	< 50 ≥ 50	58 97	15.67 ± 3.78 16.03 ± 4.58	0.617
Gender	Male Female	115 40	15.99 ± 4.20 15.61 ± 4.57	0.632
HBV infection	Absent Present	28 127	16.70 ± 4.59 15.72 ± 4.22	0.272
Liver cirrhosis	Absent Present	35 120	15.98 ± 4.00 15.87 ± 4.38	0.896
Tumor size	< 5 cm ≥ 5 cm	54 101	14.33 ± 4.52 16.73 ± 3.93	< 0.001
Tumor number	Single Multiple	62 93	15.72 ± 4.42 16.01 ± 4.22	0.679
PVTT	Absent Present	100 55	15.12 ± 4.27 17.31 ± 3.98	0.002
EHM	Absent Present	117 38	15.06 ± 4.06 18.48 ± 3.91	< 0.001
AFP (ng/mL)	< 400 ≥ 400	76 79	14.42 ± 3.89 17.33 ± 4.18	< 0.001
BCLC stage	A B C D	32 31 58 34	12.09 ± 4.02 15.31 ± 3.09 16.49 ± 3.52 19.00 ± 3.95	< 0.001

HCC: hepatocellular carcinoma; sEVs: small extracellular vesicles; HBV: hepatitis B virus; PVTT: portal vein tumor thrombus; EHM: extrahepatic metastasis; AFP: alpha-fetoprotein; BCLC: barcelona clinical liver cancer.

### Combined AFP and plasma CD147^+^ sEVs index improved diagnostic efficiency

**Figure 3:**
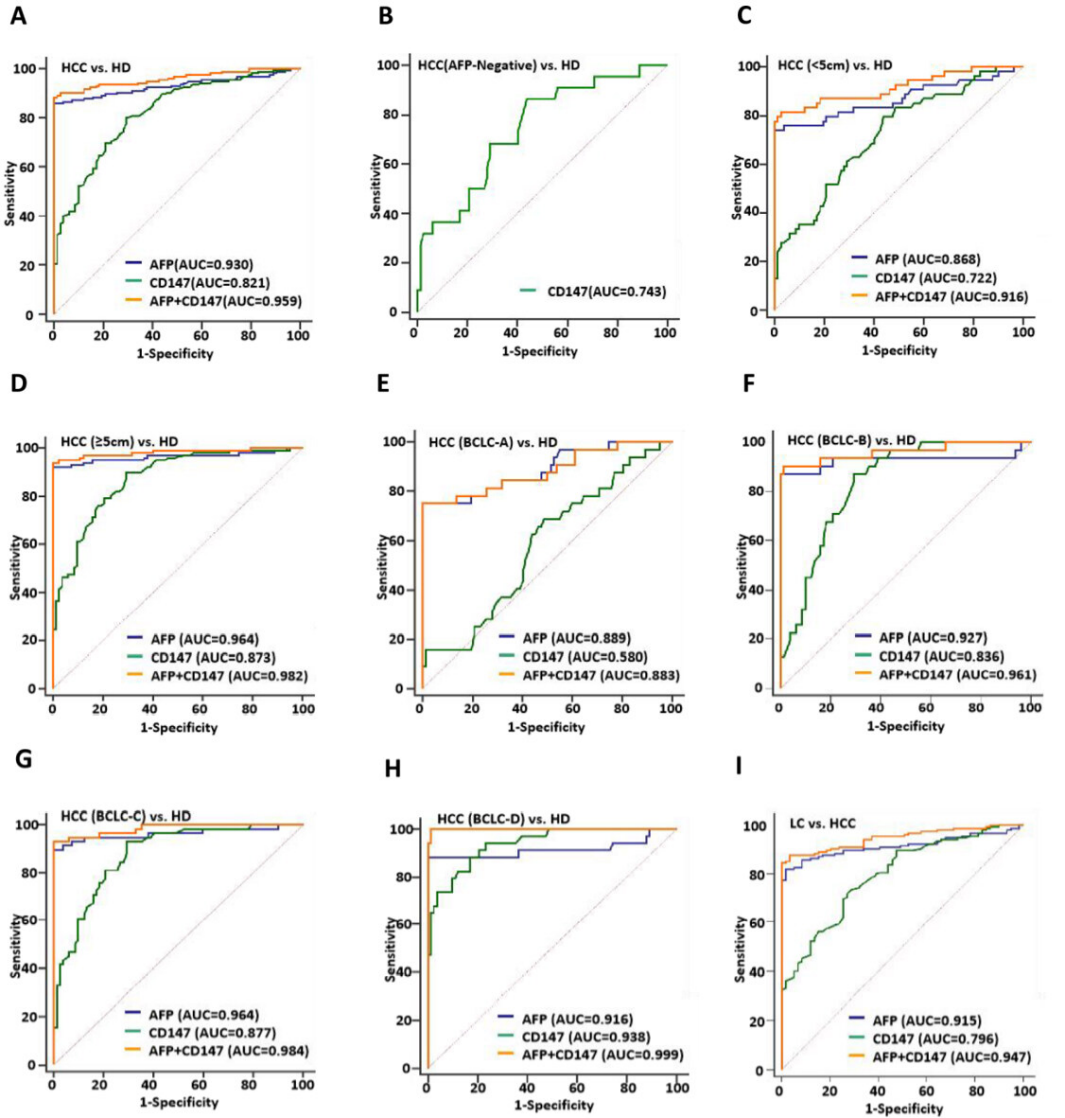
Combined index of AFP and Plasma CD147^+^ sEVs improves diagnostic efficiency for HCC. (A) The diagnostic values of CD147^+^ sEVs in distinguishing HCC patients from HD; (B) The diagnostic values of CD147^+^ sEVs for distinguishing AFP-negative HCC patients from HD; (C) The diagnostic values of CD147^+^ sEVs in distinguishing HCC patients with tumor size less than 5 cm from HD; (D) The diagnostic values of CD147^+^ sEVs in distinguishing HCC patients with tumor size greater than or equal to 5 cm from HD; (E-H): (E) The diagnostic values of CD147^+^ sEVs in distinguishing HCC patients with HCC at BCLC-A; (F)BCLC-B; (G) BCLC-C; (H)BCLC-D stage from HD; (I) The diagnostic values of CD147^+^ sEVs in distinguishing HCC patients from LC patients. HCC: hepatocellular carcinoma; CD147^+^ sEVs: CD147-positive small extracellular vesicles; HD: healthy donors; LC: liver cirrhosis; BCLC: barcelona clinical liver cancer; AFP: Alpha-fetoprotein.

We conducted ROC curve analysis to assess the diagnostic efficiency of CD147 and AFP in determining hepatic malignancy. The combined diagnostic efficiency of CD147 and AFP was higher than either marker alone [Figure 3A and Table 2]. In AFP-negative patients, CD147 diagnostic performance was effective in the diagnosis of HCC [Figure 3B]. Similarly, in terms of HCC mass size, the diagnostic efficiency of the combined CD147 and AFP index was higher than the single index, regardless of whether HCC mass was smaller or larger than 5 cm [Figure 3C and D and Table 2]. The initial diagnostic value of plasma CD147^+^ sEVs in HCC stage subgroups was also analyzed [Figure 3E-H]. Results showed that plasma CD147^+^ sEVs had poor efficacy in the diagnosis of early-stage HCC (BCLC-A stage) [Figure 3E]. In addition, we evaluated the ability of CD147 to differentiate between HCC and LC patients. We found that it was able to distinguish the two group patients favorably, and although its AUC did not reach AFP, the diagnostic performance was improved when using both CD147 and AFP in combination [Figure 3I].

**Table 2:** Main parameters of ROC curve analysis results

**Variable**	**AUC**	**95%CI**	**Sensitivity (%)**	**Specificity (%)**	**Youden index**	***P***
HCC-HD AFP CD147 AFP+CD147	0.930 0.821 0.959	0.890-0.959 0.766-0.867 0.925-0.980	85.81 80.00 88.39	100.00 70.73 100.00	0.858 0.507 0.884	< 0.001 < 0.001 < 0.001
HCC (< 5 cm) -HD AFP CD147 AFP+CD147	0.868 0.722 0.916	0.799-0.920 0.639-0.796 0.856-0.957	74.07 79.63 81.48	100.00 56.10 97.56	0.741 0.357 0.790	< 0.001 < 0.001 < 0.001
HCC (≥ 5 cm) -HD AFP CD147 AFP+CD147	0.964 0.873 0.982	0.925-0.986 0.816-0.918 0.951-0.996	92.08 90.10 94.06	100.00 70.73 100.00	0.921 0.608 0.941	< 0.001 < 0.001 < 0.001
HCC (BCLC-A)-HD AFP CD147 AFP+CD147	0.889 0.580 0.883	0.816-0.940 0.484-0.671 0.809-0.936	75.00 68.75 75.00	100.00 51.22 100.00	0.750 0.200 0.750	< 0.001 < 0.001 < 0.001
HCC (BCLC-B)-HD AFP CD147 AFP+CD147	0.927 0.836 0.961	0.862-0.967 0.755-0.899 0.907-0.989	87.10 87.10 90.32	100.00 70.73 98.78	0.871 0.578 0.891	< 0.001 < 0.001 < 0.001
HCC (BCLC-C)-HD AFP CD147 AFP+CD147	0.964 0.877 0.984	0.918-0.988 0.810-0.926 0.947-0.998	89.66 93.10 93.10	100.00 70.73 100.00	0.897 0.638 0.931	< 0.001 < 0.001 < 0.001
HCC (BCLC-D)-HD AFP CD147 AFP+CD147 LC-HD AFP CD147 AFP+CD147	0.916 0.938 0.999 0.915 0.796 0.947	0.849-0.959 0.877-0.974 0.967-1.000 0.983-1.000 0.715-0.831 0.983-1.000	88.24 88.24 100.00 86.23 78.84 92.24	100.00 82.93 98.78 100.00 86.64 100.00	0.882 0.712 0.988 0.876 0.624 0.881	< 0.001 < 0.001 < 0.001 < 0.001 < 0.001 < 0.001

ROC: receiver operating characteristic; HCC: hepatocellular carcinoma patients; HD: healthy donors; AFP: alpha-fetoprotein; BCLC: Barcelona Clinical Liver Cancer; LC: liver cirrhosis.

### CD147-dependent effect of sEVs on angiogenic activities in HUVECs *in vitro*

**Figure 4:**
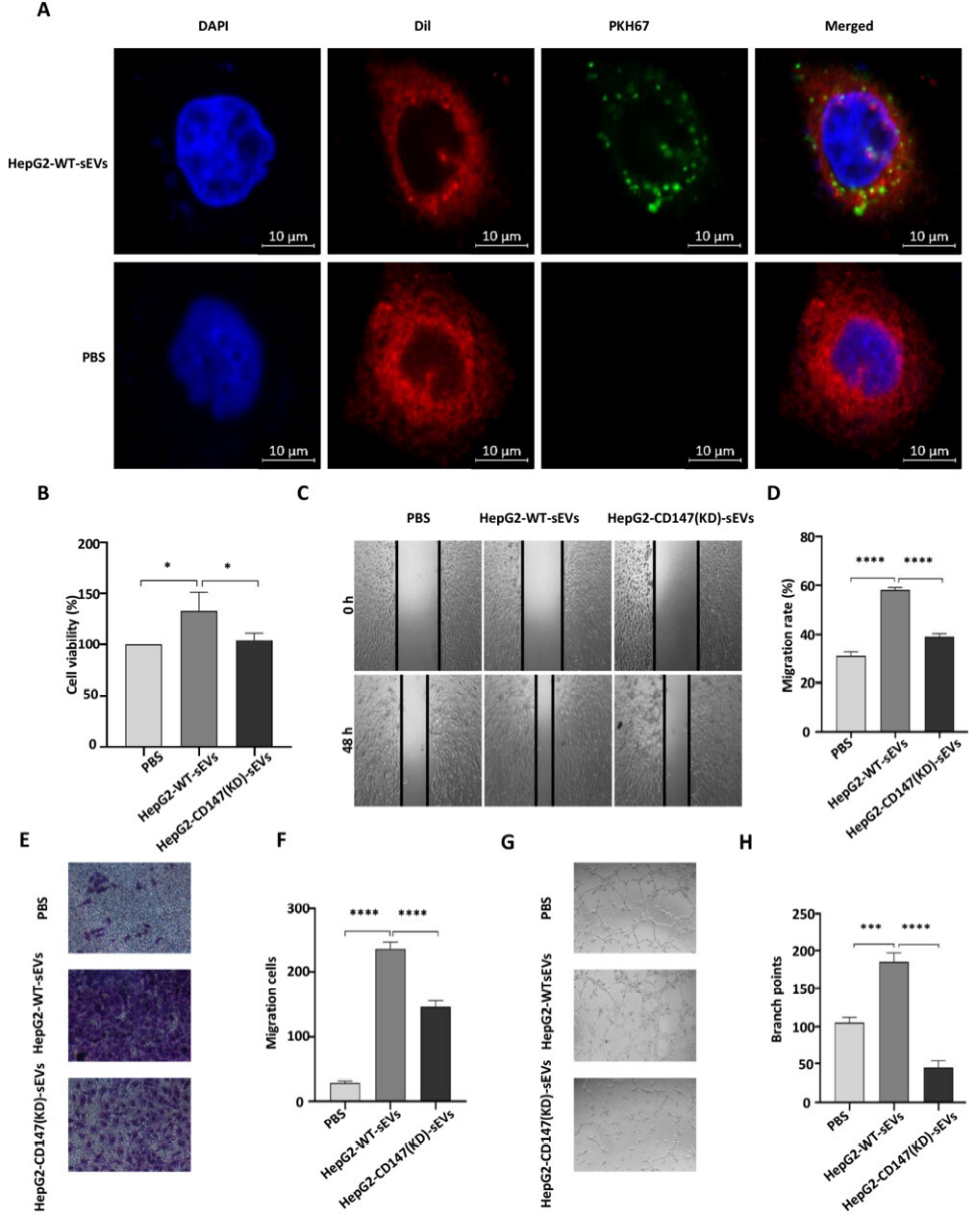
CD147 dependent effect of sEVs on angiogenic activities of HUVECs *in vitro*. (A) The uptake of PKH67-labeled HepG2-WT-sEVs by HUVECs was analyzed by immunofluorescent microscopy. Scale bar: 10 μm. Blue: DAPI; Red: Dil; Green: PKH67; (B, C, E, G): (B) HUVECs were treated with PBS, HepG2-WT-sEVs or HepG2-CD147(KD)-sEVs followed by cell proliferation assay (treatment time: 24 h); (C) wound healing assay (treatment time: 48 h); (E) transwell assay (treatment time: 24 h); (G) tube formation assay (treatment time: 6 h). Original magnification of image: × 40; (D, F, H) Quantification of wound closure as shown in (C) (D), the number of invaded cells in (E) (F), and the number of branch points in (G) (H). *: *P* < 0.05; ***: *P* < 0.001; ****: *P* < 0.0001; EVs: extracellular vesicles; HUVECs: human umbilical vein endothelial cells; PBS: phosphate‐buffered saline.

Similar to plasma-derived sEVs, we characterized sEVs purified from HepG2 culture supernatants using nFCM, TEM, and Western blotting [Supplementary Figure 3]. The level of CD147 in sEVs isolated from HepG2 cells was reduced when CD147 expression in the cells was knocked down (HepG2-CD147(KD)-sEVs) [Supplementary Figure 4A]. In addition, the percentage of CD147^+^ sEVs was also decreased in CD147 knockdown cells [Supplementary Figure 4B]. To explore the potential biological function of HepG2-derived CD147^+^ sEVs, we isolated sEVs from normal (HepG2-WT-sEVs) and CD147 knockdown (HepG2-CD147(KD)-sEVs) HepG2 cells. Following labeling with the PKH67 fluorescent dye, the sEVs were incubated with HUVECs. The labeled HepG2-WT-sEVs were efficiently taken up by HUVECs after exposure for 12 h [Figure 4A]. The sEVs isolated from normal HepG2 cells stimulated cell proliferation, migration, and invasion of HUVECs. However, the stimulatory effects were diminished when the sEVs were isolated from CD147 knockdown cells [Figure 4B-F]. This finding suggests that CD147 plays a role in the biological function of sEVs. In addition, Matrigel tube formation assay showed that HUVECs treated with HepG2-WT-sEVs had a higher number of capillary-like structures than those treated with sEVs from CD147 knocked down cells [Figure 4G and H]. This indicates that CD147 on the sEVs promotes angiogenesis.

### CD147-enriched sEVs regulated HUVEC angiogenesis through activation of the PI3K/Akt pathway

**Figure 5:**
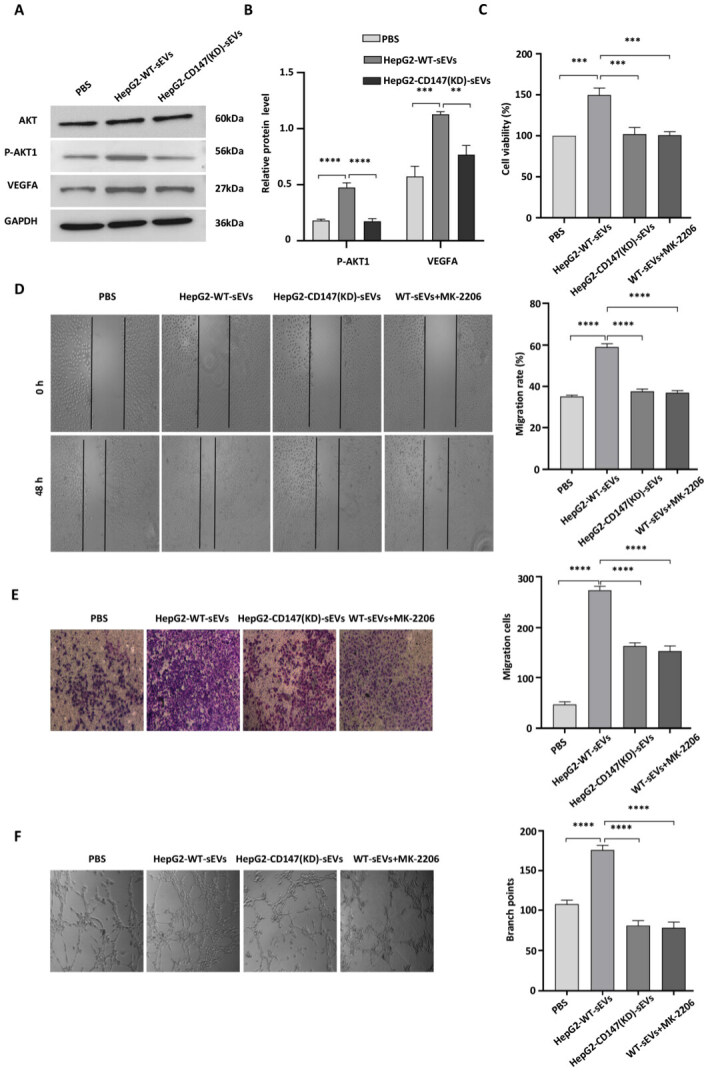
CD147-enriched sEVs regulate HUVECs angiogenesis by activating the PI3K/Akt pathway. (A, B) HUVECs were treated with sEVs for 24 h followed by western blot analysis using antibodies as indicated (A) and quantification of protein levels as shown in (A) (B). Molecular weight is indicated on the right; (C-F): (C) HUVECs were treated with PBS, HepG2-WT-sEVs, HepG2-CD147(KD)-sEVs, and MK-2206, followed by adding HepG2-WT-sEVs, respectively. The treated cells were analyzed for proliferation (treatment time: 24 h); (D) wound healing (treatment time: 48 h); (E) migration (treatment time: 24 h); (F) tube formation (treatment time: 6 h). Original magnification of image: × 40. ***: *P* < 0.001; ****: *P* < 0.0001; EVs: extracellular vesicles; HUVECs: human umbilical vein endothelial cells.

CD147-enriched-sEVs activated phosphorylated AKT1 and promoted an increase in VEGFA [Figure 5A and B]. To investigate whether HepG2-derived sEVs stimulated angiogenesis through activation of the PI3K/Akt pathway, HUVECs were treated with Akt inhibitors followed by stimulation with HepG2-WT-sEVs. The effect of sEVs on proliferation, migration, and invasion in HUVECs was blocked after treatment with the Akt inhibitor MK-2206 [Figure 5C-E]. The effect of MK-2206 on HUVECs was similar to that after stimulation with HepG2-CD147(KD)-sEVs, which suggested that CD147-enriched sEVs functioned on HUVECs through AKT1 [Figure 5C-E]. Similarly, sEV-stimulated angiogenesis was also inhibited by the Akt inhibitor and CD147 knockdown HepG2 cells-secreted sEVs [Figure 5F]. These observations indicate that HepG2-derived CD147^+^ sEVs promote angiogenesis through activation of the PI3K/Akt pathway.

### HepG2-derived CD147^+^ sEVs promoted endothelial cell angiogenesis *in vivo*

**Figure 6:**
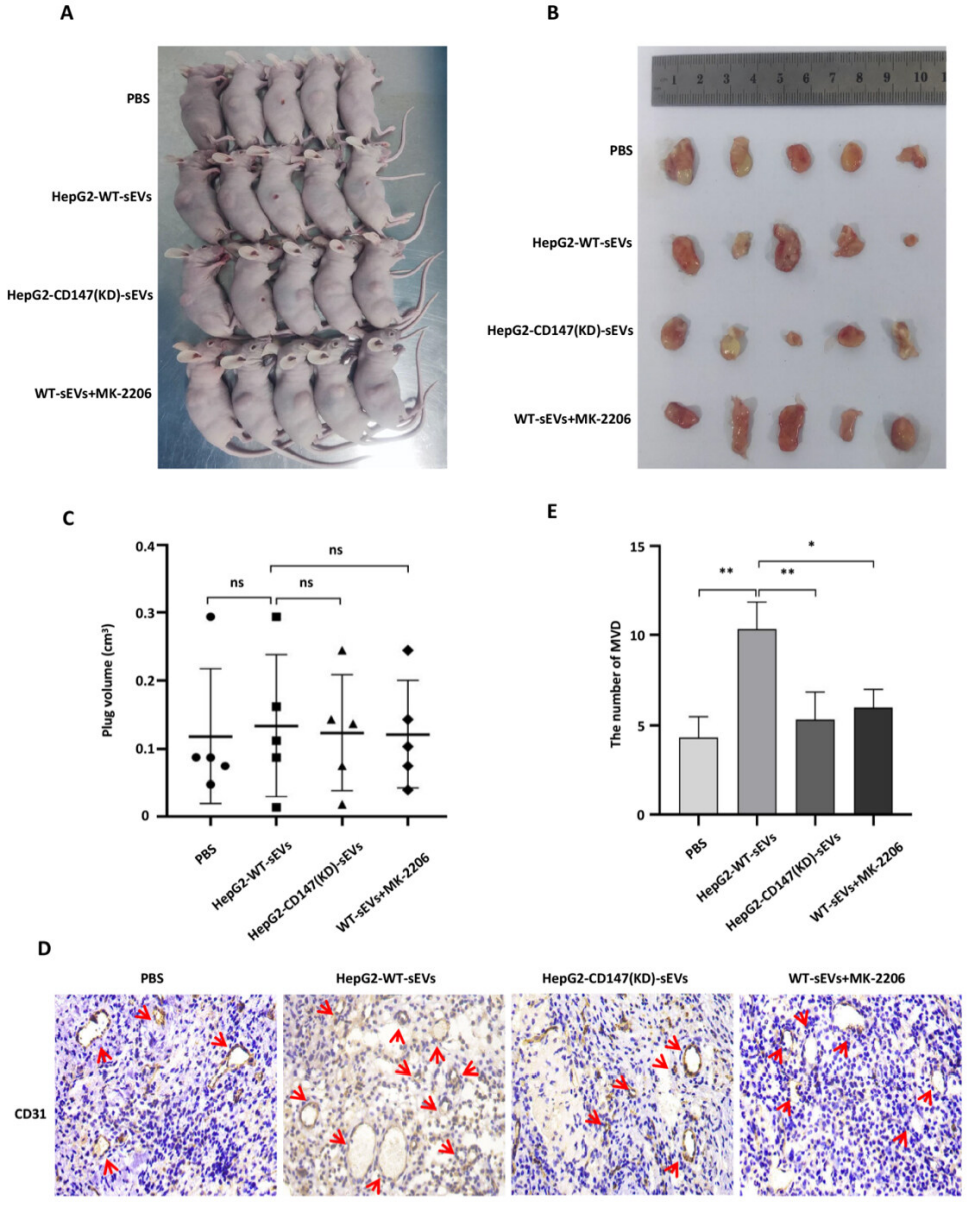
HepG2-derived CD147^+^ sEVs promote endothelial cell angiogenesis *in vivo*. (A) HUVECs treated with PBS, HepG2-WT-sEVs, HepG2-CD147(KD)-sEVs, and MK-2206 together with HepG2-WT-sEVs were injected subcutaneously into female BALB/c nude mice. Mice were then euthanized and photographed 14 days after injection; (B) Matrigel plugs were excised and photographed 14 days after subcutaneous injection; (C) Volume of matrix plugs in each group from (B); (D) Matrigel plugs were subjected to immunohistochemistry to examine the expression of CD31 in endothelial cells. Original magnification of image: × 100; (E) Quantification of the number of MVD in each group. MVD: microvessel density; ns: no significance; *: *P* < 0.05; **: *P* < 0.01; EVs: extracellular vesicles; HUVECs: human umbilical vein endothelial cells; PBS: phosphate‐buffered saline.

To evaluate whether HepG2-derived CD147^+^ sEVs also exert a stimulatory effect on micro-vessel formation *in vivo*, we performed Matrigel plug assays, a classic murine model used to examine the angiogenic activity of endothelial cells. The results showed no significant difference in plug volume between the four groups [Figure 6A-C]. Microvessel density (MVD) index CD31 immunohistochemical staining revealed a significant increase in MVD number in the HepG2-derived CD147^+^ sEVs group in comparison to the PBS and HepG2-CD147(KD)-sEVs groups [Figure 6D and E]. In addition, this was reduced in the MK-2206 treatment group [Figure 6D and E].

### Plasma levels of CD147^+^ sEVs are associated with angiogenesis

**Figure 7:**
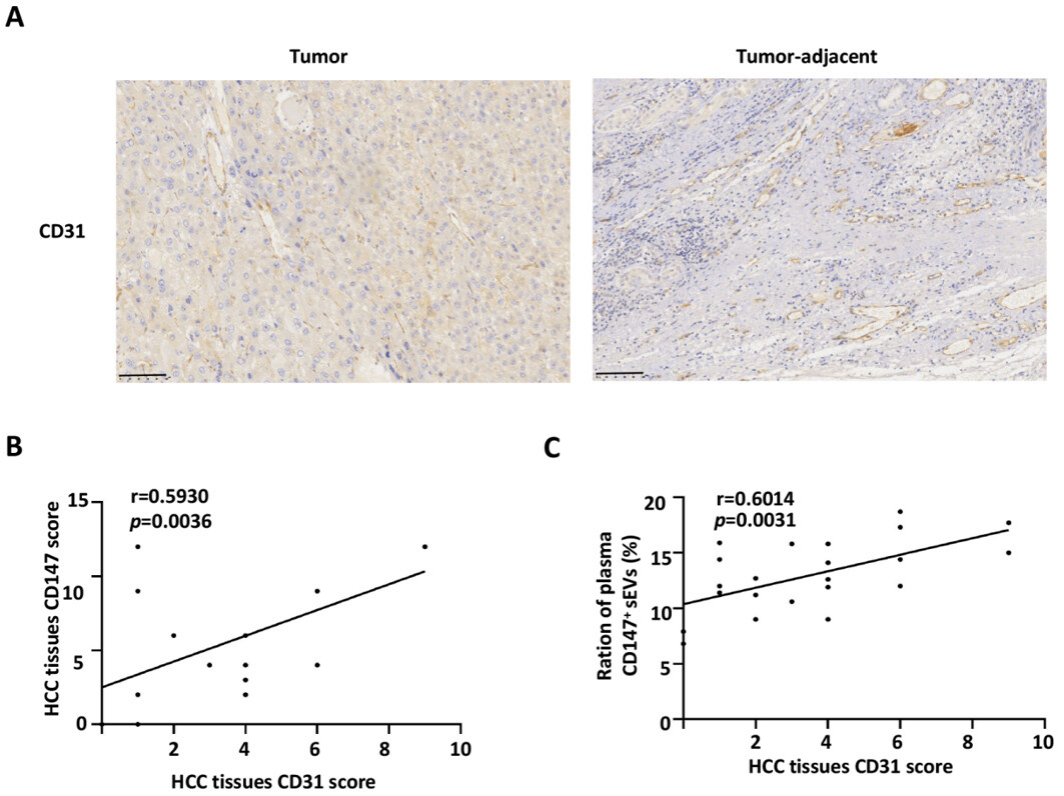
Plasma levels of CD147^+^ sEVs are associated with angiogenesis. (A) Tumor and tumor-adjacent samples were subjected to immunohistochemistry to examine the expression of CD31 (*n* = 22). Representative images are shown. Scale bar: 100 μm; (B) Correlation between CD31 and CD147 expression in HCC tissues (*n* = 22); (C) Correlation between CD31 expression in HCC tissues and the ration of plasma CD147^+^ sEVs (*n* = 22). HCC: hepatocellular carcinoma; CD147^+^ sEVs: CD147-positive small extracellular vesicles.

To investigate the relationship between plasma CD147^+^ sEVs and tumor angiogenesis, immunohistochemistry was used to detect CD31 expression in the patient tumors. This analysis reflects endothelial angiogenesis. The analysis showed that the expression of CD31 was elevated in patients with high levels of plasma CD147^+^ sEVs [Figure 1D and 7A]. In addition, CD31 expression was positively correlated with tissue CD147 and plasma CD147^+^ sEVs expression [Figure 7B and C].

## DISCUSSION

CD147 is overexpressed in many cancer types and plays a key role in oncogenesis and cancer metastasis^[21,22]^. It has recently been demonstrated that CD147 stimulates the expression of VEGF in both the tumor and stromal compartments^[23]^. In the current study, we found that plasma CD147^+^ sEVs expression was upregulated in HCC patients. The CD147^+^ sEVs level was positively correlated with CD31 expression in patient tissues. A combination of the CD147 and AFP indices significantly improved the diagnostic efficiency in HCC compared with the use of a single marker alone. Unfortunately, the diagnostic performance of CD147^+^ sEVs alone is inferior to that of AFP. This discrepancy could potentially be attributed to specific characteristics of patient cohorts in different regions, sample size, potential confounders, *etc*., all of which may affect diagnostic accuracy. Serum AFP is widely recognized and used for HCC diagnosis^[24]^. However, it is difficult to characterize HCC with a single biomarker, since it is a complex disease caused by various risk factors with multiple pathogenic mechanisms^[25]^. 30%-40% of all patients with HCC are AFP-negative. It is difficult to provide appropriate methods of diagnosis and assessment of treatment for such a cohort so far^[26,27]^. Notably, sEVs-associated CD147 was found to be a good diagnostic surrogate in AFP-negative patients. Since there are limitations in both sensitivity and specificity when using single protein as a biomarker, the combination of CD147, AFP, and potentially other clinical indices/biomarkers may improve diagnostic efficiency and stage determination in HCC.

The development and progression of cancer result from the synergistic action of tumor cells and neighboring cells, including fibroblasts and endothelial cells^[28,29]^. In this regard, alterations in the stromal microenvironment, such as angiogenesis, modified extracellular matrix composition, and unbalanced protease activity, are essential regulatory factors in tumor growth and invasion. In previous studies, evidence showed that the PI3K/Akt pathway is an important signaling pathway in the process of angiogenesis^[30,31]^. This pathway regulates a wide spectrum of cellular processes including cell proliferation, survival, growth, and motility^[32,33]^. Tumor-derived CD147 is reportedly essential to the activation of the PI3K-Akt pathway in tumor cells^[16]^. In our study, we found that HepG2-derived CD147^+^ sEVs stimulated HUVEC proliferation, migration, invasion, and angiogenesis. Furthermore, we showed that CD147^+^ sEVs upregulated Akt phosphorylation, suggesting that activation of the PI3K/Akt pathway may underlie the stimulatory activity of sEVs during cell proliferation and angiogenesis. However, our preliminary data cannot rule out the possibility that other important signaling molecules are involved in the function of CD147 in activated HUVECs.

### Limitations of the study

While our study has uncovered intriguing insights, it is important to acknowledge its limitations. The sample size utilized in evaluating the diagnostic potential of CD147+ sEVs for HCC was modest. Expanding the sample size would enhance the robustness and reliability of our findings. Currently, the isolation of sEVs presents challenges in terms of time and cost, which must be addressed to facilitate their practical application in clinical diagnostics for HCC. In our mechanistic investigations, we demonstrated that CD147+ sEVs can stimulate angiogenesis in HUVECs via the PI3K/AKT pathway. However, the involvement of signaling molecules upstream and downstream of CD147 in this process has not been explored. Further research is necessary to fully elucidate the mechanisms.

In summary, we found that sEVs released from hepatocellular carcinoma cells may induce pro-angiogenic activity in HUVECs through activation of the PI3K/Akt pathway. Our study supports that plasma-derived CD147^+^ sEVs could be used as a diagnostic marker for HCC detection, especially in AFP-negative HCC patient cohorts.

## DECLARATIONS

### Availability of data and materials

The datasets used and/or analyzed during the current study are available from the corresponding author upon reasonable request.

### Copyright

© The Author(s) 2023.
