# Supplementary material for: Hepatocellular carcinoma cell-derived small extracellular vesicle-associated CD147 serves as a diagnostic marker and promotes endothelial cell angiogenesis via the PI3K/Akt pathway
Source: Extracell Vesicles Circ Nucl Acids. 2023 Oct 12;4(4):532–47. doi: 10.20517/evcna.2023.30 (PMC12066417; doi:10.20517/evcna.2023.30)
Supplement: Supplementary file 1 [file evcna-4-4-532-SupplementaryMaterials.pdf]

Hepatocellular carcinoma cell-derived small extracellular vesicle-associated CD147 serves as a diagnostic marker and promotes endothelial cell angiogenesis via the PI3K/Akt pathway

De-Fa Huang[1,#], Wen-Juan Zhang[1,2,#], Jie Chen[1,3], Zhi-Gang Jiao[1], Xiao-Ling Wang[1], Ding-Yu Rao[4], Wei-Song Li[5], Die Hu[1], Fang-Fang Xie[1], Xiao-Xing Wang[6], Zheng-Zhe Li[6], Xiao-Mei Yi[6], Ji-Yang Wu[6], Yu Jiang[7], Qi Wang[6], Tian-Yu Zhong[1,2]

[1]Laboratory Medicine, First Affiliated Hospital of Gannan Medical University, Ganzhou 341000, Jiangxi, China.

[2]Precision Medicine Center, First Affiliated Hospital of Gannan Medical University, Ganzhou 341000, Jiangxi, China.

[3]Department of Clinical Laboratory, Yongchuan Hospital of Chongqing Medical University, Chongqing 402160, Sichuan, China.

[4]Department of Cardiothoracic Surgery, First Affiliated Hospital of Gannan Medical University, Ganzhou 341000, Jiangxi, China.

[5]Department of pathology, First Affiliated Hospital of Gannan Medical University, Ganzhou 341000, Jiangxi, China.

[6]The First School of Clinical Medicine, Gannan Medical University, Ganzhou 341000, Jiangxi, China.

[7]Department of Pharmacology and Chemical Biology, University of Pittsburgh School of Medicine, Pittsburgh, PA 15260, USA.

[#]These authors contributed equally and considered joint first authors.

Correspondence to: Dr. Tian-Yu Zhong, Laboratory Medicine, First Affiliated Hospital of Gannan Medical University, No. 128, Jinling West Road, Ganzhou 341000, Jiangxi, China. E-mail: zhongtianyu@gmail.com

How to cite this article: Huang DF, Zhang WJ, Chen J, Jiao ZG, Wang XL, Rao DY,

Li WS, Hu D, Xie FF, Wang XX, Li ZZ, Yi XM, Wu JY, Jiang Y, Wang Q, Zhong TY. Hepatocellular carcinoma cell-derived small extracellular vesicle-associated CD147 serves as a diagnostic marker and promotes endothelial cell angiogenesis via the PI3K/Akt pathway. *Extracell Vesicles Circ Nucleic Acids* 2023;4:432-47.
http://dx.doi.org/10.20517/evcna.2023.30

Supplementary figure legends

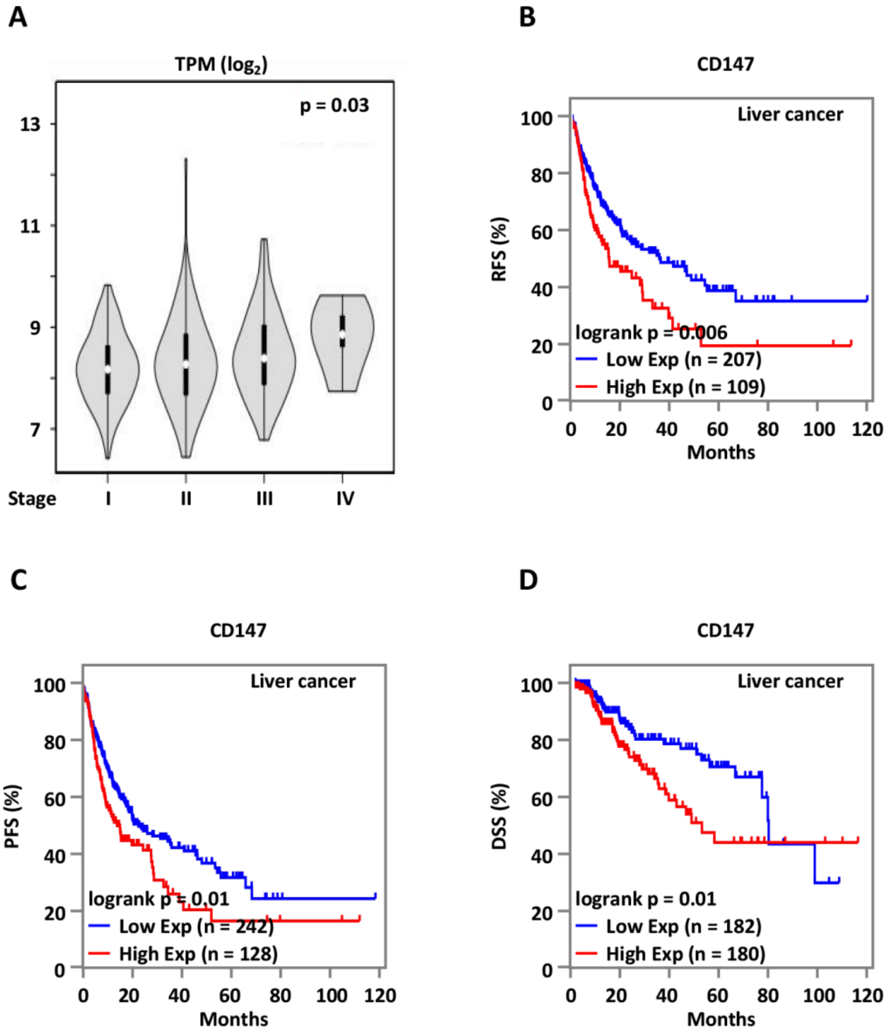

Supplementary Figure 1. High levels of CD147 are associated with liver tumorigenesis. Related to Figure 1. (A) The pathological stage plot (TPM, log2) of CD147 in a cohort of clinical liver cancer (n=369) samples from TCGA; (B-D) Kaplan Meier survival analyses for RFS (relapse-free survival) (B), PFS (progression-free survival) (C), and DSS (disease-specific survival) (D) of liver cancer using CD147 as input.

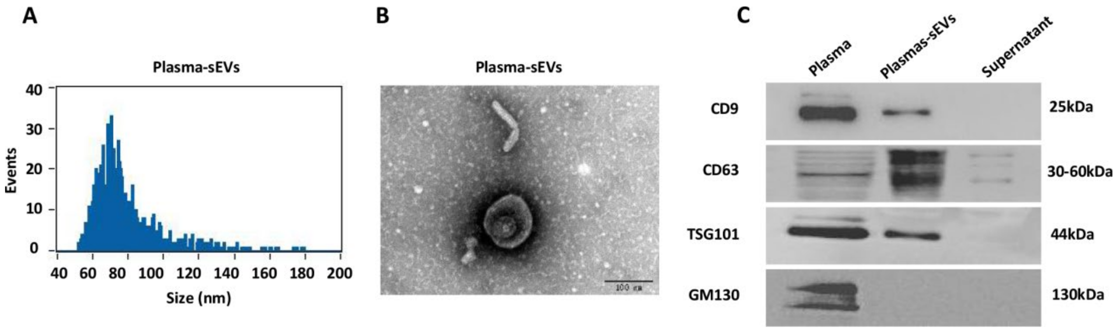

Supplementary Figure 2. Characterization of plasma-derived sEVs. Related to Figure 2. (A) Size of sEVs was measured by nano-flow cytometer (nFCM) analysis; (B) sEVs were captured by transmission electron microscope (TEM), Scale bar: 100 µm; (C) Plasma, plasma-sEVs and supernatant were subjected to western blotting analysis, showing the presence of protein markers commonly associated with sEV subpopulations, e.g., CD9, CD63, and TSG101. GM130 (the Golgi protein) indicates that non-EV cellular components in cells.

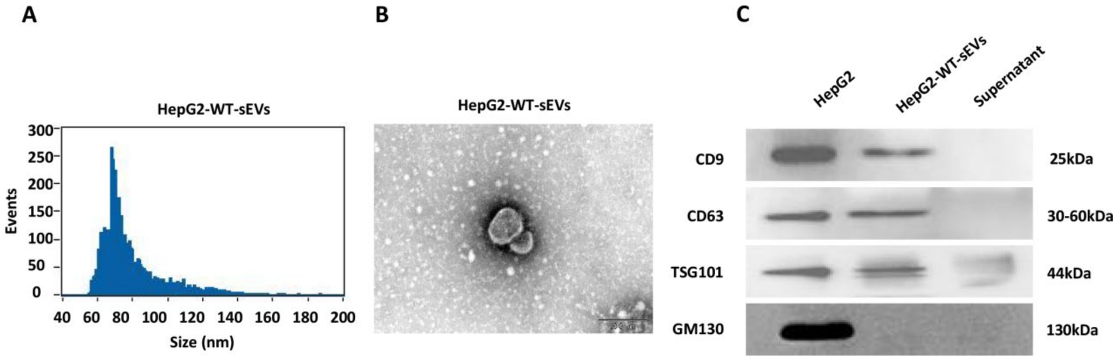

Supplementary Figure 3. Characterization of HepG2-derived sEVs. Related to Figure 4. (A) Size of sEVs was measured by nano-flow cytometer (nFCM) analysis; (B) sEVs were captured by transmission electron microscope (TEM), Scale bar: 100 µm; (C) HepG2 cells, HepG2-WT-sEVs, and supernatant were subjected to western blotting analysis, showing the presence of protein markers commonly associated with sEV subpopulations, e.g., CD9, CD63, and TSG101. GM130 (the Golgi protein) indicates that non-EV cellular components in cells.

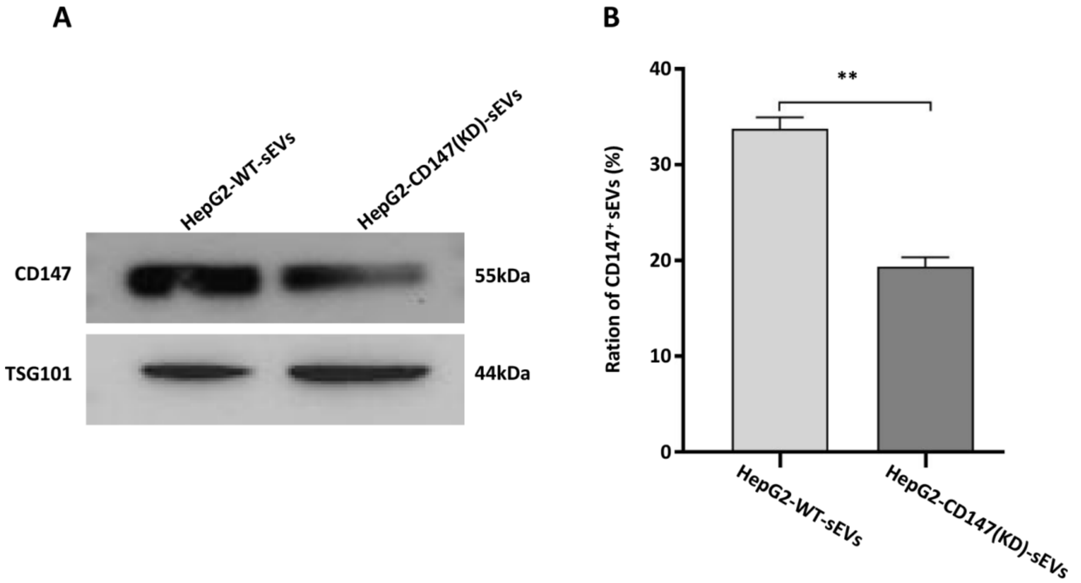

Supplementary Figure 4. CD147 Stable Knockdown sEVs purified from HepG2 culture supernatants. Related to Figure 4. (A) sEVs from HepG2 cells infected with lentiviral shRNA specifically targeting CD147 (CD147 KD) were followed by western blotting using antibodies as indicated. Molecular weight is indicated on the right; (B) The related levels of $CD147^{+}$ sEVs isolated from culture supernatant of wild type (WT) or CD147 knockdown HepG2 cells (**, $P < 0.01$).

Supplementary Tables

Supplementary Table 1. Original information of healthy donors (HD)

NO.	Gender	Age (years)	CD147+sEVs (%)	AFP (ng/ml)
1	Male	<50	8.4	1.79
2	Female	≥50	5.2	1.87
3	Male	<50	10.6	3.99
4	Male	<50	13.2	2.16
5	Male	<50	11.5	4.78
6	Male	<50	13	5.09
7	Female	<50	9.8	3.42
8	Male	<50	10.1	2.59
9	Male	<50	13.1	1.32
10	Male	≥50	9.1	2.68

11	Female	<50	19.9	3.22
12	Male	<50	13.4	2.25
13	Male	<50	10.7	1.99
14	Female	≥50	12.2	3.68
15	Male	<50	11.2	2.00
16	Male	≥50	11.1	0.96
17	Male	≥50	6.6	2.03
18	Male	<50	0.8	5.24
19	Female	≥50	7.4	1.44
20	Female	<50	14.3	1.66
21	Female	≥50	13.5	2.16
22	Male	<50	15.2	1.58
23	Female	≥50	12.8	3.66
24	Female	<50	12	1.63
25	Male	<50	15.1	2.80
26	Male	<50	12.4	5.38
27	Female	<50	11.9	1.53
28	Female	<50	13.2	2.55
29	Female	<50	14.7	3.51
30	Male	≥50	8.1	1.23
31	Male	<50	9.4	2.11
32	Male	<50	13.7	1.39
33	Female	<50	16.3	1.90
34	Female	<50	8.9	1.12
35	Female	<50	9.6	4.09
36	Female	<50	14.9	2.32
37	Female	<50	8.6	4.83
38	Female	<50	12	2.74
39	Male	<50	9.9	1.59

40	Male	<50	10.8	4.32
41	Female	<50	16.3	1.69
42	Male	<50	14.1	2.53
43	Female	<50	6.9	2.64
44	Male	<50	9.3	4.9
45	Female	<50	7.8	4.58
46	Female	<50	10.1	1.34
47	Male	<50	9.2	2.64
48	Male	<50	8.9	3.02
49	Female	<50	4.5	3.55
50	Male	<50	3.9	2.45
51	Female	<50	10.4	1.54
52	Male	≥50	12.1	2.00
53	Female	<50	10.5	2.91
54	Female	<50	10.5	1.53
55	Female	<50	10.5	4.14
56	Female	<50	6.1	1.52
57	Male	<50	12.3	3.32
58	Female	<50	7.4	2.01
59	Female	<50	10.4	7.90
60	Male	<50	3.6	3.66
61	Male	≥50	12.4	6.89
62	Male	<50	7.3	2.07
63	Female	<50	11.3	2.36
64	Male	≥50	11	2.68
65	Female	<50	9.2	3.68
66	Female	<50	9.5	1.12
67	Male	<50	15.8	1.92
68	Female	<50	16.6	2.76

69	Male	<50	8.9	2.19
70	Female	<50	7.7	4.42
71	Female	<50	17.8	1.07
72	Female	<50	16.6	4.73
73	Female	<50	13.8	2.6
74	Male	≥50	17.3	1.92
75	Female	≥50	7	7.54
76	Male	≥50	6.2	3.41
77	Female	<50	10.3	2.92
78	Female	<50	8	4.50
79	Male	<50	14.7	2.16
80	Female	<50	8.9	1.98
81	Male	<50	6	1.05
82	Female	<50	9.6	2.10

Supplementary Table 2. Original information of liver cirrhosis (LC) patients

NO.	Gender	Age (years)	CD147+sEVs (%)	AFP (ng/ml)
1	Male	<50	14.8	1.25
2	Male	≥50	3.7	31.9
3	Male	<50	14.7	2.23
4	Female	≥50	3.8	2.63
5	Female	≥50	4.5	7.33
6	Male	<50	13.0	3.64
7	Male	<50	9.7	5.33
8	Male	≥50	13.3	4.07
9	Male	<50	9.8	3.7
10	Female	<50	12.7	1.56
11	Male	≥50	10.9	3.13
12	Male	<50	6.9	1.8

13	Male	≥50	9.1	4.57
14	Male	≥50	9.4	4.48
15	Male	<50	15.7	2.62
16	Female	≥50	10	14.9
17	Female	<50	14.9	2.2
18	Male	≥50	12.2	1.81
19	Female	≥50	17.8	1.59
20	Male	<50	16.8	2.24
21	Female	≥50	13.1	3.31
22	Male	<50	10.8	2.2
23	Male	<50	8.9	3.02
24	Female	≥50	5.8	3.24
25	Female	<50	12.4	2.75
26	Male	≥50	6.1	2.85
27	Female	≥50	7.8	6.26
28	Male	<50	10.8	1.72
29	Female	<50	9.7	0.98
30	Female	≥50	8.2	3.58
31	Female	≥50	4.7	3.56
32	Male	≥50	10.6	2.03
33	Male	≥50	9.6	2.6
34	Male	<50	12.6	11.5
35	Female	≥50	17.3	2.65
36	Female	≥50	10.5	2.84
37	Male	≥50	9	1.64
38	Female	<50	11.8	3.24
39	Female	≥50	11	2.75
40	Male	<50	15.7	10.60
41	Female	≥50	14.4	2.29

42	Male	≥50	15.2	4.58
43	Male	<50	14.2	4.79
44	Male	<50	10.9	3.61
45	Male	≥50	12.8	7.59
46	Male	≥50	13	1.51
47	Male	≥50	16.1	1.53
48	Male	<50	11.3	1.39
49	Female	≥50	10.4	4.38
50	Male	<50	14.4	14.5
51	Male	<50	17.4	3.81
52	Male	<50	12.2	2.92
53	Male	<50	14.4	3.58
54	Female	≥50	2.9	1.86
55	Male	<50	10.3	1.96
56	Female	≥50	8.8	2.61
57	Female	<50	10.9	1.32
58	Female	≥50	12.8	2.44
59	Male	≥50	3.9	3.76

Supplementary Table 3. Original information of hepatocellular carcinoma (HCC) patients

No	Age (Years)	Gender	HBV infection	Liver Cirrhosis	Tumor size	Tumor number	Portal vein tumor thrombus（PVTT）	Extrahepatic Metastasis (EHM)	Barcelona Clinical Liver Cancer(BCLC)	AFP（ng/ml）	Preoperation CD147^{+}sEVs（%）	Postoperation CD147^{+}sEVs（%）
1	⩾50	Male	Absent	Present	≥5cm	Single	Present	Absent	B	80568	15.6	
2	⩾50	Female	Present	Present	≥5cm	Multiple	Absent	Absent	C	3572	21.9	
3	⩾50	Male	Absent	Present	≥5cm	Multiple	Absent	Absent	B	1320.5	23.5	16.7
4	⩾50	Male	Present	Absent	≥5cm	Single	Absent	Absent	C	501	19.7	
5	＜50	Female	Present	Absent	＜5cm	Single	Absent	Absent	C	6.87	19.7	
6	＜50	Male	Present	Absent	≥5cm	Single	Absent	Absent	C	10906	21.9	
7	⩾50	Male	Present	Absent	≥5cm	Single	Absent	Absent	C	382	15.5	
8	⩾50	Male	Present	Absent	≥5cm	Multiple	Present	Present	D	56855	15.1	
9	＜50	Male	Present	Absent	≥5cm	Single	Absent	Absent	A	48.5	13.2	12
10	＜50	Male	Absent	Present	≥5cm	Single	Absent	Absent	C	96.9	12.8	

11	⩾50	Male	Present	Present	≥5cm	Single	Absent	Absent	C	382	15.5	
12	＜50	Male	Absent	Present	≥5cm	Multiple	Present	Present	D	256	13.6	
13	＜50	Female	Present	Absent	≥5cm	Multiple	Present	Present	B	145	14.9	
14	＜50	Male	Present	Absent	≥5cm	Single	Absent	Absent	C	47.1	17.5	
15	＜50	Male	Absent	Present	≥5cm	Single	Absent	Absent	A	96.9	12.8	
16	⩾50	Male	Absent	Absent	≥5cm	Single	Absent	Absent	A	1411	22.7	
17	⩾50	Male	Absent	Absent	≥5cm	Multiple	Absent	Absent	C	72.7	16.2	
18	＜50	Male	Absent	Present	≥5cm	Single	Absent	Absent	B	1.21	14.2	13.8
19	⩾50	Female	Absent	Absent	＜5cm	Single	Absent	Absent	B	2654	16.1	
20	⩾50	Male	Present	Present	≥5cm	Multiple	Present	Absent	C	484000	14.2	
21	＜50	Male	Absent	Absent	≥5cm	Multiple	Present	Absent	C	1827	22.3	
22	⩾50	Male	Absent	Present	＜5cm	Single	Present	Present	C	2654	16.6	
23	⩾50	Male	Absent	Present	≥5cm	Single	Absent	Absent	B	382	15.5	
24	⩾50	Female	Present	Absent	≥5cm	Multiple	Present	Absent	C	354	15.6	
25	⩾50	Male	Absent	Absent	≥5cm	Multiple	Present	Absent	D	2.89	18.7	

26	⩾50	Male	Absent	Present	≥5cm	Single	Absent	Absent	D	1.41	22.7	
27	⩾50	Male	Present	Present	≥5cm	Multiple	Absent	Absent	D	1124.5	20.2	
28	＜50	Female	Present	Absent	＜5cm	Single	Absent	Absent	A	3.3	13.6	12.7
29	⩾50	Male	Present	Present	≥5cm	Multiple	Absent	Absent	C	122	14.1	
30	⩾50	Female	Present	Absent	≥5cm	Multiple	Present	Absent	B	443	20.5	
31	＜50	Male	Present	Present	＜5cm	Single	Absent	Present	D	44277	14.2	
32	＜50	Male	Present	Absent	≥5cm	Single	Absent	Absent	B	25598	14.7	13.6
33	＜50	Male	Present	Absent	≥5cm	Single	Present	Absent	C	138	16.2	
34	⩾50	Male	Absent	Absent	＜5cm	Single	Absent	Absent	D	1200	12	
35	＜50	Male	Absent	Absent	≥5cm	Single	Absent	Absent	C	154	17.5	14
36	⩾50	Male	Present	Absent	≥5cm	Multiple	Present	Present	A	484000	12	
37	＜50	Male	Present	Present	≥5cm	Single	Absent	Absent	C	1860	14.9	
38	⩾50	Female	Present	Absent	＜5cm	Multiple	Absent	Present	D	8688	10.6	
39	⩾50	Male	Present	Present	＜5cm	Multiple	Absent	Absent	A	267	18.1	16
40	⩾50	Male	Present	Present	＜5cm	Multiple	Absent	Absent	A	79.9	9.5	

41	＜50	Male	Present	Present	≥5cm	Multiple	Present	Absent	C	132597	18.4	
42	＜50	Male	Present	Present	≥5cm	Multiple	Absent	Absent	C	2708.6	21.7	
43	＜50	Female	Present	Present	＜5cm	Multiple	Absent	Absent	C	79.3	13.9	
44	＜50	Male	Absent	Present	＜5cm	Single	Absent	Absent	B	46794	15.7	
45	⩾50	Female	Present	Present	≥5cm	Multiple	Present	Absent	C	894	11.5	
46	⩾50	Male	Present	Present	＜5cm	Multiple	Present	Absent	C	2.12	16.4	
47	⩾50	Male	Present	Present	＜5cm	Multiple	Present	Absent	A	128	9.9	
48	＜50	Female	Present	Present	≥5cm	Multiple	Present	Present	D	484000	14.2	12.3
49	⩾50	Male	Present	Present	≥5cm	Multiple	Present	Present	D	405.8	19.1	
50	⩾50	Male	Absent	Absent	≥5cm	Multiple	Present	Present	D	1404	25.4	
51	＜50	Male	Present	Present	≥5cm	Multiple	Present	Present	D	4506	18.4	
52	＜50	Male	Present	Absent	＜5cm	Multiple	Absent	Absent	B	153674	14.1	
53	＜50	Female	Present	Present	≥5cm	Multiple	Present	Absent	C	80	14.9	
54	＜50	Male	Absent	Absent	＜5cm	Multiple	Present	Absent	C	56	12.7	
55	＜50	Male	Absent	Present	≥5cm	Multiple	Absent	Absent	B	26	20.6	

56	≥50	Male	Absent	Present	≥5cm	Multiple	Absent	Absent	B	1666	17.2	14.8
57	＜50	Male	Present	Present	≥5cm	Multiple	Present	Present	D	3987	20.4	
58	≥50	Female	Present	Present	＜5cm	Multiple	Absent	Absent	A	18.1	8.6	
59	＜50	Male	Present	Present	≥5cm	Multiple	Absent	Absent	C	1118	21.7	
60	＜50	Male	Present	Present	＜5cm	Single	Absent	Absent	B	699	11.9	
61	≥50	Female	Present	Absent	≥5cm	Multiple	Present	Present	D	14320	21.9	17.2
62	≥50	Male	Present	Absent	≥5cm	Single	Absent	Absent	B	205616	16.4	
63	≥50	Female	Present	Present	≥5cm	Multiple	Present	Present	D	59.1	15.7	
64	≥50	Male	Present	Present	≥5cm	Single	Present	Absent	C	29.8	17.4	
65	＜50	Male	Present	Present	＜5cm	Multiple	Absent	Absent	C	5095	14.4	
66	≥50	Female	Present	Present	≥5cm	Single	Present	Absent	C	9900	18.6	
67	＜50	Male	Present	Present	＜5cm	Multiple	Present	Present	D	4506	18.4	
68	＜50	Male	Present	Absent	≥5cm	Multiple	Absent	Absent	B	62971	14.1	
69	≥50	Male	Present	Absent	＜5cm	Single	Absent	Absent	A	3.6	6.8	
70	＜50	Male	Present	Present	≥5cm	Single	Absent	Absent	C	39.4	12.6	

71	＜50	Female	Present	Present	≥5cm	Multiple	Present	Present	D	217132	13.3	
72	＜50	Male	Present	Present	≥5cm	Multiple	Absent	Absent	A	8613	11.5	
73	⩾50	Male	Present	Present	≥5cm	Single	Present	Absent	C	23.1	18.5	14.3
74	⩾50	Female	Absent	Absent	＜5cm	Single	Absent	Absent	A	2.25	11.2	
75	⩾50	Female	Present	Present	≥5cm	Multiple	Present	Absent	B	35.9	12.8	
76	⩾50	Male	Absent	Present	≥5cm	Multiple	Absent	Absent	A	10.2	11.3	
77	＜50	Male	Present	Present	≥5cm	Single	Present	Absent	C	436	14.4	
78	⩾50	Male	Present	Present	≥5cm	Multiple	Present	Absent	C	195	18.4	12.9
79	⩾50	Male	Present	Present	＜5cm	Single	Absent	Absent	C	318	13.1	
80	＜50	Female	Present	Present	≥5cm	Single	Absent	Absent	C	4.7	12.8	
81	＜50	Male	Present	Present	≥5cm	Single	Absent	Absent	C	96.9	10.5	
82	⩾50	Male	Present	Present	＜5cm	Single	Absent	Absent	A	4.02	12.9	
83	＜50	Female	Present	Present	＜5cm	Single	Absent	Absent	A	2232	8.6	
84	＜50	Male	Absent	Present	＜5cm	Multiple	Absent	Absent	B	1.09	11.1	
85	⩾50	Female	Absent	Absent	≥5cm	Single	Absent	Present	D	45.4	15.6	

86	＜50	Male	Present	Present	≥5cm	Single	Present	Absent	C	5	19.7	
87	＜50	Female	Present	Present	≥5cm	Single	Absent	Absent	C	93.8	14.7	
88	＜50	Male	Present	Present	≥5cm	Multiple	Present	Absent	C	42	13.7	
89	⩾50	Male	Present	Present	≥5cm	Single	Absent	Absent	A	656	20.2	15
90	⩾50	Male	Present	Present	＜5cm	Multiple	Absent	Absent	A	21765	10.6	
91	⩾50	Male	Present	Present	＜5cm	Multiple	Present	Present	D	533	21.3	
92	＜50	Female	Present	Present	＜5cm	Single	Present	Present	D	1.9	19.8	
93	＜50	Male	Present	Present	≥5cm	Single	Absent	Present	C	103652	18.4	
94	⩾50	Male	Present	Present	＜5cm	Single	Absent	Absent	A	5395	13.7	
95	＜50	Male	Present	Present	≥5cm	Single	Present	Absent	C	672	25.8	
96	⩾50	Female	Present	Present	＜5cm	Single	Absent	Absent	A	1078	20.2	
97	⩾50	Male	Present	Present	≥5cm	Single	Absent	Absent	A	318	4.9	
98	⩾50	Male	Present	Present	≥5cm	Multiple	Present	Absent	C	372	15.5	
99	⩾50	Female	Present	Absent	≥5cm	Multiple	Absent	Absent	B	4.41	13	
100	⩾50	Male	Present	Present	≥5cm	Single	Absent	Present	D	1960	22.9	14.6

101	⩾50	Male	Present	Present	≥5cm	Multiple	Present	Present	D	92.7	20.5	
102	⩾50	Male	Present	Present	＜5cm	Multiple	Absent	Absent	A	142	11.1	
103	⩾50	Male	Present	Present	＜5cm	Multiple	Absent	Absent	B	15.2	12.1	
104	＜50	Male	Present	Present	＜5cm	Multiple	Absent	Absent	A	2.44	11.3	
105	⩾50	Male	Present	Present	≥5cm	Multiple	Absent	Present	D	1200	19.4	17.1
106	⩾50	Male	Present	Present	＜5cm	Single	Absent	Absent	C	93.5	17.6	
107	⩾50	Female	Present	Present	＜5cm	Multiple	Absent	Absent	A	5395	13.7	
108	⩾50	Male	Present	Present	＜5cm	Single	Absent	Absent	C	286	13.1	
109	⩾50	Male	Present	Present	≥5cm	Multiple	Present	Present	D	678.4	20.5	
110	⩾50	Female	Present	Present	＜5cm	Multiple	Absent	Absent	A	2.35	10.3	
111	＜50	Female	Present	Present	＜5cm	Single	Present	Absent	C	65.3	8.1	
112	⩾50	Female	Absent	Present	≥5cm	Single	Absent	Absent	C	1210	15.4	14.3
113	⩾50	Male	Present	Present	＜5cm	Multiple	Absent	Absent	A	142	11.1	
114	⩾50	Female	Present	Present	≥5cm	Multiple	Present	Present	D	3862	20.5	
115	⩾50	Male	Present	Present	≥5cm	Multiple	Present	Absent	B	372	13.2	

116	⩾50	Male	Present	Present	≥5cm	Multiple	Absent	Present	D	1210	17.6	
117	⩾50	Female	Present	Present	≥5cm	Multiple	Absent	Absent	C	552	21.4	
118	＜50	Male	Present	Present	≥5cm	Multiple	Absent	Absent	B	774	14.9	
119	⩾50	Male	Present	Present	＜5cm	Multiple	Absent	Present	C	1.38	13.6	
120	⩾50	Female	Present	Present	≥5cm	Multiple	Absent	Absent	B	23.6	12.5	
121	⩾50	Male	Present	Present	＜5cm	Single	Absent	Absent	B	142	13.1	
122	⩾50	Male	Present	Present	≥5cm	Multiple	Present	Absent	C	1641	17.1	
123	＜50	Male	Present	Present	≥5cm	Multiple	Present	Absent	C	379690	18.1	16.8
124	＜50	Male	Present	Present	≥5cm	Multiple	Absent	Absent	C	516	17.7	
125	⩾50	Female	Present	Present	＜5cm	Single	Present	Present	D	8.41	23.7	
126	⩾50	Male	Present	Present	≥5cm	Multiple	Absent	Absent	C	43.8	12	
127	⩾50	Male	Present	Present	≥5cm	Multiple	Absent	Absent	C	4350.6	21.4	
128	＜50	Male	Present	Present	≥5cm	Multiple	Present	Present	D	484000	21.4	17.8
129	⩾50	Male	Present	Present	≥5cm	Multiple	Absent	Present	D	32054	25	
130	⩾50	Female	Present	Present	≥5cm	Multiple	Absent	Present	D	8.72	17	

131	⩾50	Female	Absent	Present	＜5cm	Single	Present	Present	D	1.45	27.3	
132	⩾50	Male	Present	Present	＜5cm	Single	Absent	Absent	A	858	7.9	
133	⩾50	Male	Present	Present	≥5cm	Multiple	Present	Absent	C	4509	15	14.6
134	⩾50	Male	Present	Absent	＜5cm	Single	Absent	Present	B	1386	13.6	
135	＜50	Male	Present	Present	≥5cm	Multiple	Present	Absent	C	2.79	12.7	
136	⩾50	Male	Present	Present	＜5cm	Single	Absent	Absent	A	10.4	18	
137	＜50	Female	Present	Present	≥5cm	Single	Absent	Absent	C	34241	15.8	
138	⩾50	Male	Present	Present	＜5cm	Multiple	Absent	Present	D	2341.5	21.3	
139	⩾50	Male	Present	Present	≥5cm	Multiple	Present	Absent	B	528	14.4	
140	⩾50	Male	Present	Present	＜5cm	Multiple	Absent	Absent	B	3.9	17.8	
141	⩾50	Male	Present	Absent	≥5cm	Multiple	Present	Present	D	38231	16.9	
142	⩾50	Male	Present	Present	≥5cm	Multiple	Absent	Absent	B	884	15.5	
143	⩾50	Male	Present	Present	＜5cm	Multiple	Present	Present	C	22.3	18.7	
144	⩾50	Male	Present	Present	＜5cm	Multiple	Absent	Present	D	734	21.3	
145	⩾50	Male	Present	Present	≥5cm	Multiple	Absent	Absent	B	1343	15.9	

146	＜50	Male	Present	Present	≥5cm	Multiple	Absent	Absent	A	12	10.8
147	＜50	Female	Present	Present	＜5cm	Multiple	Present	Present	B	72394	22.1
148	⩾50	Male	Present	Present	≥5cm	Multiple	Absent	Absent	C	11744	23.5
149	⩾50	Male	Present	Present	≥5cm	Multiple	Absent	Absent	B	32.1	10.3
150	⩾50	Female	Present	Present	≥5cm	Multiple	Absent	Absent	A	1.89	9
151	⩾50	Male	Present	Present	＜5cm	Multiple	Absent	Absent	A	466.9	7.5
152	⩾50	Female	Present	Present	≥5cm	Multiple	Absent	Absent	C	667	13.6
153	⩾50	Male	Absent	Absent	＜5cm	Single	Absent	Absent	A	2.56	11.4
154	＜50	Male	Present	Present	＜5cm	Multiple	Absent	Absent	A	9.22	12.4
155	＜50	Male	Present	Present	≥5cm	Single	Absent	Absent	B	582	17.3
